# 

**Published:** 1871-03

**Authors:** 


					﻿Vol. XII. MARCH, 1871.	No. .3'
CHICAGO
Medical Examiner,
N. S. DAVIS, M.D., Editor,
AND
F. H. Davis, Assist.-Editor.
TERMS, 83.00 I»ER ANNUM, irv ADλAME.
CHICAGO:
ROBERT FERGUS’ SONS, PRINTERS,
12 CLARK AND 242—248 ILLINOIS STREET
1871.
				

